# Knowledge, use and practices of licensed dietitians related to dietary supplements in Lebanon

**DOI:** 10.1017/S136898002100495X

**Published:** 2022-07

**Authors:** Cyrille Nacouzi, Vanessa Tarazi, Lara Kabalane, Maya Hosry, Mira Hleyhel

**Affiliations:** 1 Nutrition Department, Faculty of Public Health II, Lebanese University, Fanar, Lebanon; 2 INSPECT-LB, Institut National de Santé Publique, Epidémiologie Clinique et Toxicologie, Beirut, Lebanon; 3 CERIPH, Center for Research in Public Health, Pharmacoepidemiology Surveillance Unit, Faculty of Public Health, Lebanese University, Fanar, Lebanon

**Keywords:** Dietary supplements, Knowledge score, Recommendation, Licensed dietitians, Use

## Abstract

**Objective:**

To evaluate Lebanese licensed dietitians’ knowledge, prevalence of use and recommendation of dietary supplements (DS), and their associated factors.

**Design:**

Cross-sectional survey.

**Setting:**

Dietitians across Lebanon were contacted through the telephone and were asked to participate in the study. An online self-administered questionnaire was designed and sent to dietitians either by email or by WhatsApp, between 4 March and 4 May 2020.

**Participants:**

This study included 319 dietitians randomly selected from the dietitians’ list that was provided by the Lebanese Ministry of Public Health.

**Results:**

Around 75 % of dietitians had a knowledge score above 50 %. Overall, 73·7 % of them have used DS and 46·1 % have recommended them. Higher knowledge score was associated with less years of experience, using scientific articles as source of information on DS, and participating in research. Resorting to pharmacists was significantly associated with both nutrient supplements (NS) and herbal supplements (HS) use, whereas referring to health food stores was associated with HS use only. NS recommendation to patients was associated with personal NS use (OR = 3·38, *P* < 0·001), considering pharmacists as a source of information on DS (OR = 2·29, *P* = 0·01) and discussing DS with patients (OR = 3·82, *P* = 0·01). Having personally used HS (OR = 12·23, *P* < 0·001) and having discussed DS with patients (OR = 8·51, *P* = 0·01) increased the likelihood of recommending HS.

**Conclusions:**

A proper DS education, the elaboration of national scientific guidelines and the implementation of concise laws regarding the regulations of DS would play a crucial role in supporting dietitians’ practices and improving the quality of patient care with respect to DS.

The popularity of complementary and alternative medicine (CAM) has increased around the world the last decades and has become of great importance to today’s healthcare consumers, practitioners, researchers and industries. In the last decade, approximately 40 % of the adults in the USA^(1)^, 10 to 40 % of the general population in Europe^(2)^, and between 30 and 80 % of adults in the Middle East^(3–6)^ reported the use of some form of CAM to aid with a variety of health conditions such as diabetes, hypertension, weight loss and pain management.

As a prominent part of CAM therapies, dietary supplements (DS) are commonly consumed around the world. In fact, with the international rise of DS use, the DS global market expanded to reach 109 billion dollars in 2015^(7)^ and projections estimated it to grow to 272·4 billion dollars by 2028^(8)^.

Among the general population, the prevalence of DS use ranged from 22 % to 52 %: 52 % in the USA^(9)^, 43·2 % in Australia^(10)^, 15 % in men and 24 % in women in the United Kingdom, 40 % in Canada, 24 % in men and 46 % in women in France and 22 % in men and 33 % in women in Sweden^(11)^. In the Middle East, although population-level data are still not available for many countries, studies conducted on specific groups showed that 37·8 % of Dubai residents^(12)^, 65 % of university students in Egypt^(13)^, 33·1 % of university students in Saudi Arabia^(14)^ and 71·4 % of adults in Kuwait^(15)^ have reported the use of DS, with multivitamins, vitamins C and D being among the most consumed DS.

This overall increase in the use of DS observed within different populations has occurred despite little evidence for their effectiveness and the observation of adverse effects. Furthermore, consumers are often exposed to a vast amount of misinformation about supplements that exist on different platforms such as the internet, magazines and even on the supplement itself. Therefore, when considering the use of a DS, seeking the advice of a healthcare professional is recommended^(16,17)^.

Dietitians, as members of the healthcare team, have the potential to improve individuals’ nutritional status and are routinely involved in assessing energy and macronutrients’ needs, as well as vitamins and minerals’ requirements for each patient. Based on patient’s needs and intakes, dietitians may propose the use of a specific DS. Backed up by scientific data, they are a reliable source of DS information and are likely to know more about products, their potential effects and the interactions that should be avoided^(18)^. Studies on dietitians showed that their level of knowledge regarding DS varied across countries, and poor to good levels of DS-related knowledge were reported^(19,20)^. While the prevalence of DS use in dietitians ranged between approximately 18 % and 84 %, prevalence rates of DS recommendation by dietitians to patients varied between approximately 27 % and 97 %^(19–27)^ depending on the country.

In Lebanon, a Mediterranean country with distinct dietary patterns, different health concerns and different DS market, the prevalence of DS use was reported to be approximately 35 % among the general population in 2015^(28)^. The most popular DS used were herbal supplements (HS) such as anise tea, green tea and Zhourat which is a blend of wildflowers, herbs, leaves, and fruits consumed as tea, vitamins and minerals’ supplements and products labelled as natural health products^(28,29)^. While only 5·8 % of users have received DS information from their healthcare providers, the majority has been informed by their friends and family^(29)^.

To the best of our knowledge, no studies have been conducted on the knowledge, use and practices of dietitians in Lebanon regarding DS. Therefore, the objectives of this study were to assess the knowledge of licensed dietitians (LD) in Lebanon regarding DS and to evaluate the prevalence of personal use and that of recommending DS to patients. The types of supplements, the reasons and barriers for their use and recommendation were assessed. In addition, the factors likely influencing their knowledge, personal use and willingness to recommend supplements were identified.

## Methods

### Study design and population

A cross-sectional study was conducted on all LD enlisted in the Ministry of Public Health (MoPH) in Lebanon. The target population included all the LD who were directly consulting with patients and/or clients at the time of their participation in the study. Therefore, the dietitians included in this study were those who worked in hospitals, diet centres, dispensaries and private clinics on all Lebanese territories. Whereas dietitians who only worked in the education, community, food safety and non-nutrition fields, dietetic students, unemployed or retired LD and dietitians working abroad were excluded from the study.

**Fig. 1:**
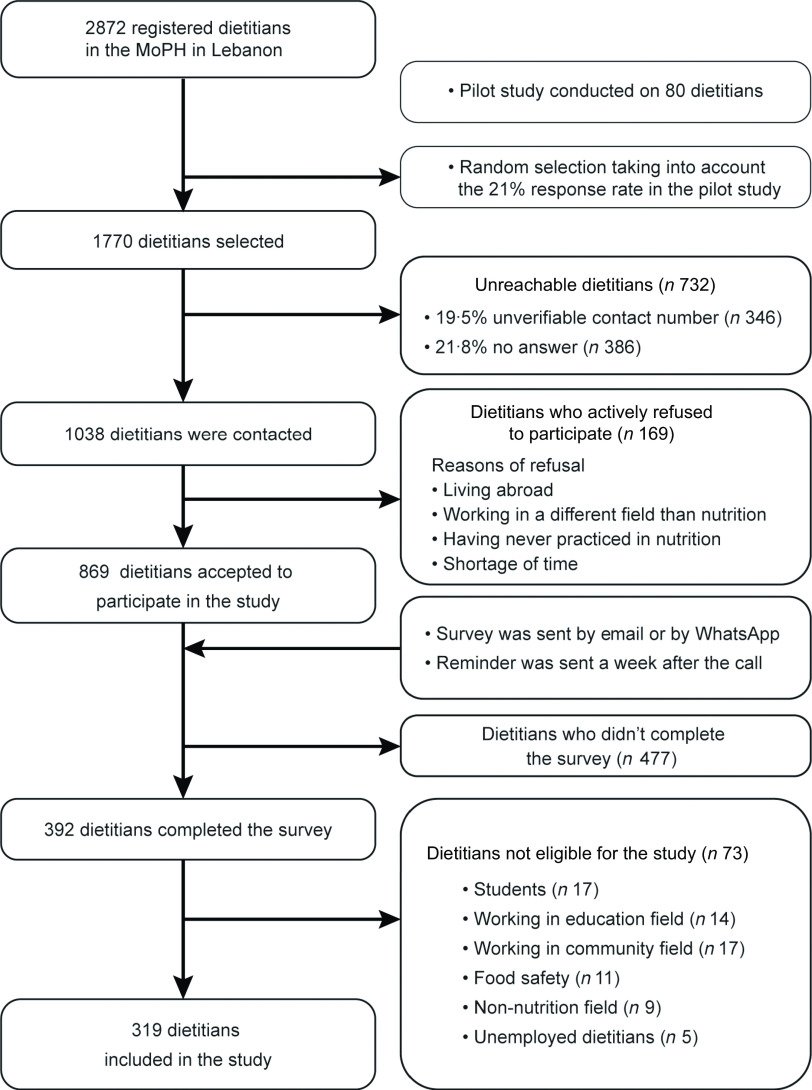
Flowchart of the study participants

A pilot study was conducted on eighty dietitians to determine the expected prevalence of use which was 53 % and was used to calculate the sample size with a 95 % confidence level and 5 % margin of error. The estimated sample size was 338 and was increased by a factor of 1/(1-0·8) in order to take into account the dietitians who refused to participate and those who could not be reached. Therefore, a simple random sample of 1770 dietitians was selected from the MoPH list, using IBM SPSS Statistics 25 software (Fig. 1).

### Survey instrument

An English self-administered questionnaire was designed based on the literature review^(20,25,26,29–32)^ to test the knowledge of LD regarding DS and to assess the use and practices of LD towards DS in Lebanon. For this research, DS were divided into two categories: (1) nutrient supplements (NS) including vitamins, minerals, multivitamins and non-vitamins non-mineral supplements; and (2) HS including capsules and tea bags. The questionnaire included four sections. The first section comprised questions about dietitians’ sociodemographic characteristics (gender, age, educational level, field and years of practice), anthropometric measurements (weight and height), physical activity level, and tobacco and alcohol use. The second and third sections assessed dietitians’ personal use and practices towards both NS and HS. Dietitians were asked about the types of DS used and recommended, as well as the reasons and barriers for DS use and recommendation. The last section tested dietitians’ knowledge regarding DS with a validated test developed by Steyn *et al.*, having fifty questions covering the following concepts: (a) absorption, bioavailability and bioconversion of micronutrients; (b) deficiency and potential toxicity of micronutrients; (c) recommended intake of micronutrients; and (d) structure and functions of micronutrients^(20)^. Questions about dietitians’ sources of information regarding DS were also included.

The survey was hosted online on Google Forms and was tested by two expert dietitians. Slight modifications were made considering their comments in order to improve the consistency and understandability of questions.

### Data collection

Selected dietitians were contacted by telephone and were asked to participate in the study. Following their agreement over the phone, a link to the online questionnaire was sent either by email or by WhatsApp. Only one response was allowed per participant. A week after the call, a message was sent to the dietitians who had not completed the questionnaire at the time to remind them to complete it, to improve the participation rate. All data were collected by four licensed dietetic students under the supervision of an advisor, between 4 March and 4 May 2020.

### Statistical analysis

Descriptive statistics were performed to present dietitians’ sociodemographic characteristics, knowledge scores, personal use and practices including recommendation and sale of DS, using frequencies and percentages for categorical variables and means and standard deviations for continuous variables. For the knowledge score, participants were given one point for each correct answer and zero points for each wrong or ‘I do not know’ answers. For each participant, score of correct answers was summed (0 to 50) and converted into percentage (0 to 100) as a representative of total knowledge score. NS use and HS use were defined as having personally consumed at least one NS and at least one HS, respectively, in the last 12 months. NS recommendation and HS recommendation were defined as having recommended to patients at least one NS and at least one HS, respectively, in the last 12 months.

To identify factors associated with knowledge score, personal use of DS and with DS recommendation to patients, bivariate analyses were conducted using Pearson’s correlation to test the relationship between two continuous variables, *χ* ^2^ and Fisher’s exact tests to compare proportions for categorical variables, independent sample *t* test to compare means for dichotomous variables and one-way ANOVA or Kruskal–Wallis test to compare means for polytomous variables. Factors associated with DS total knowledge score were identified using a multiple linear regression model. Four multivariate logistic regression models were used to identify variables associated with personal use and recommendation of both NS and HS separately. All variables that have shown a *P*-value <0·2 in the bivariate analysis were included in the multivariate models. During the regression analysis process, multicollinearity checking was considered using the variance inflation factor. For the four logistic regression models, all variance inflation factors lied between 1 and 2, therefore no multicollinearity diagnostic was detected. A *P*-value <0·05 was considered statistically significant. Data were processed and analysed using the Statistical Package for Social Sciences software, version 25.0.

## Results

### Demographic and lifestyle characteristics

Of the 1038 dietitians contacted by telephone, 9·5 % actively refused to participate and the survey was therefore sent to 869 dietitians. A total of 392 subjects completed the questionnaire with a participation rate of 37·7 %. After excluding students, retired or unemployed dietitians, and those working in the community, food safety, education and non-nutrition fields, 319 dietitians were included in the study (Fig. 1).

Most dietitians were females and had an average age of 28 years. More than half of them had a bachelor’s degree and 4·5 years of practice on average, with the majority working in a private practice setting mainly in Beirut and Mount Lebanon (Table 1).

**Table 1:** Characteristics of the dietitians included in the study (*n* 319)

	Mean	sd
Age (years)	28·06	4·53
Years of practice (years)	4·52	3·16
Missing values (*n* 17)		

	Frequency	%
Gender		
Male	5	1·6
Female	314	98·4
Level of education		
Bachelor’s degree	177	55·5
Postgraduate degree	142	44·5
Field of practice		
Clinical-based (hospital)	47	14·7
Private practice, diet centre and dispensary	229	71·8
Clinical-based and private practice	43	13·5
Workplace by governorates of Lebanon		
Beirut	82	25·7
Mount Lebanon	101	31·7
Beqaa	26	8·2
North Lebanon	47	14·7
South Lebanon	42	13·2
More than one governorate	18	5·6
Missing values (*n* 3)		
BMI (kg/m^2^)		
Underweight (<18·5)	24	7·5
Normal weight (18·5 to <25)	258	80·9
Overweight/obese (≥25)	37	11·6
Physical activity level		
Lightly active (30 min, 1–3 d/week or less)	138	43·3
Moderately active (45 min, 3–5 d/week)	142	44·5
Very active (1 h, 6–7 d/week or more)	39	12·2
Smoking		
Non-smoking	283	88·7
Smoking	36	11·3
Alcohol consumption		
Never	181	56·7
Occasionally	138	43·3

Summary statistics are expressed as mean and standard deviation for continuous variables and as frequency and percentage for categorical variables.

### Sources of information regarding dietary supplements

Dietitians reported scientific articles (70·5 %) as the most frequently used source of DS information, followed by international guidelines (65·2 %), former college education (58 %), former trainings and workshops (36·4 %), and pharmacists (20·7 %). More than the half of them (56·7 %) did not attend any campaign on DS (education campaigns that focus on the health benefits, risks, safety and the responsible use of DS) and only 20 % of them have participated in research about DS.

### Knowledge regarding dietary supplements

Dietitians’ mean knowledge score was 60·5 over 100, with only 10 % of participants having poor knowledge (score <35 %), 14 % having scored below average (score between 36 and 50 %), 30 % having good knowledge (score between 51 and 65 %) and 46 % having very good knowledge regarding DS (score >65 %). The highest score was for concept D, relating to dietitians’ knowledge of micronutrients’ structure and function, whereas LD scored the lowest on questions related to micronutrients’ absorption and bioavailability (concept A) (Table 2).

**Table 2:** Knowledge score, and prevalence of personal use and recommendation practices regarding DS among dietitians in Lebanon (*n* 319)

Knowledge regarding dietary supplements	Mean	sd
Total knowledge score	60·49	17·32
Concepts* subscores		
Concept A subscore	55·50	18·07
Concept B subscore	62·81	19·23
Concept C subscore	60·81	19·67
Concept D subscore	64·52	28·37

Personal use of dietary supplements	Frequency	%
Prevalence of DS use	235	73·7
Prevalence of NS use	226	70·8
Prevalence of HS use	56	17·6

Recommendation of dietary supplements		
Prevalence of DS recommendation	147	46·1
Prevalence of NS recommendation	126	39·5
Prevalence of HS recommendation	53	16·6

DS, dietary supplement; NS, nutrient supplement; HS, herbal supplement.

* Concept A, absorption and bioavailability of micronutrients; concept B, deficiency and toxicity of micronutrients;

Concept C, recommended intakes of micronutrients; concept D, structure and functions of micronutrients.

All concepts had a minimum subscore of 0. Maximum subscores were 92·86, 94·44, 100 and 100 for concepts A, B, C and D, respectively.

Results of the multiple linear regression for the factors associated with knowledge score regarding DS are presented in Table 3. Total knowledge score was independently associated with the years of work experience (*β* = 4·41, *P* = 0·02), using scientific articles as source of information on DS (*β* = 7·23, *P* = 0·001), and participating in research (*β* = 5·43, *P* = 0·02) (Table 3).

**Table 3:** Multiple linear regression for the factors associated with knowledge score regarding dietary supplements of dietitians in Lebanon (*n* 302)

	Dietary supplements knowledge				
	*β* †	Standardised *β*	Lower 95 % CI of *β*	Upper 95 % CI of *β*	*P*-value
Years of practice	4·41	0·12	0·56	8·26	0·02*
Smoking	3·99	0·07	−2·07	10·05	0·19
Sources of DS information					
Former college education	3·20	0·09	−0·67	7·08	0·10
Scientific articles	7·23	0·18	2·85	11·60	0·001*
International guidelines	2·47	0·06	−1·69	6·64	0·24
Participating in research about DS	5·43	0·12	0·69	10·17	0·02*
Using HS	−4·8	−0·10	−9·96	0·36	0·07
Discussing DS with patients	4·21	0·09	−0·90	9·33	0·10

DS, dietary supplement; HS, herbal supplement.

* A *P*-value <0·05 is considered statistically significant.

† *β*, regression coefficient.

The analysis included the following variables: years of practice, smoking, sources of DS information (former college education, scientific articles and international guidelines), participating in research about DS, using HS and discussing DS with patients.

### Personal use of dietary supplements

Overall, the prevalence of DS use by dietitians in Lebanon was 73·7 %, among which 70·8 % reported that they have used at least one NS in the last 12 months (Table 2) with vitamin D being the most used NS. Only 4·1 % of vitamin D users were regular users and 42·3 % were occasional or frequent users. Other commonly used NS included Mg (38·9 %), Fe (33·2 %), vitamin C (32·3 %), multivitamins + Fe (30·4 %), prebiotics/probiotics (29·2 %) and *n*-3 and *n*-6 fatty acids (26·6 %). Most dietitians using Fe supplements (36·8 %) were daily users, whereas most Mg (48·4 %), vitamin C (51·5 %) and pre/probiotics (65·6 %) consumers were occasional users (see online Supplemental Table 1). As for HS, only 17·6 % of dietitians reported their use in the last 12 months. Green tea (8·5 %) and ginger (7·8 %) were the most used capsules, while green tea (13·2 %), chamomile (9·4 %), ginger (9·4 %) and peppermint (7·5 %) were the most used teas. Most HS users reported an occasional consumption of these supplements in the last 12 months (see online Supplemental Table 2).

Dietitians reported filling nutrient gaps (30·2 %), skin, hair and nails health (26·8 %) and overall health (23·8 %) as main reasons for using DS. Among other reasons mentioned, bone health was reported by 20·4 % of the dietitians, while women’s health and gastrointestinal health accounted, respectively, for 20 % and 18·7 % of dietitians’ reasons of DS use. Among non-users, the main barriers for the use of DS were their lack of interest (10·3 %), and their lack of confidence (7 %) and training (continuing education workshops, courses and conferences) (2·6 %) regarding DS. Concerns regarding both potential negative effects (7·7 %) and regulation (7 %) of DS were also reported.

Results of the multiple logistic regression for the factors associated with personal use of DS are presented in Table 4. Smoking decreased the likelihood of using NS (OR = 0·38, *P* = 0·02). In addition, learning about DS from pharmacists and from attending campaigns was associated with increased odds of using NS (OR = 2·68, *P* = 0·01 and OR = 2·06, *P* = 0·01, respectively). Whereas receiving information regarding DS from health food stores (shops primarily selling organic foods, health foods, local produce and nutritional supplements) and participating in research increased the likelihood of HS use (OR = 3·71, *P* = 0·03 and OR = 2·10, *P* = 0·04, respectively).

**Table 4:** Multiple logistic regression for the factors associated with using nutrient supplements (*n* 302) and herbal supplements (*n* 319) by dietitians in Lebanon

	Nutrient supplements use†				Herbal supplements use			
	OR	Lower 95 % CI	Upper 95 % CI	*P*-value	OR	Lower 95 % CI	Upper 95 % CI	*P*-value
Age (years)	1·03	0·95	1·12	0·42	–	–	–	–
Years of practice	1·08	0·95	1·22	0·21	–	–	–	–
Level of education								
Bachelor’s degree	1	–	–	–	–	–	–	–
Postgraduate degree	1·10	0·64	1·90	0·71				
Field of practice								
Clinical-based	1	–	–	–	–	–	–	–
Private practice, diet centre and dispensary	1·23	0·57	2·62	0·59				
Clinical-based and private practice	1·82	0·60	5·53	0·41				
BMI (kg/m^2^)								
Overweight/obese (≥25)	–	–	–	–	1	–	–	–
Underweight (<18·5)					–	–	–	0·99
Normal (18·5 to <25)					1·02	0·41	2·55	0·95
Smoking								
Non-smoking	1	–	–	–	–	–	–	–
Smoking	0·38	0·16	0·87	0·02*				
Alcohol consumption								
Never	–	–	–	–	1	–	–	–
Occasionally					0·56	0·29	1·07	0·08
Sources of DS information								
Former college education								
No	1	–	–	–	–	–	–	–
Yes	1·68	0·98	2·90	0·06				
Former trainings/workshops								
No	–	–	–	–	1	–	–	–
Yes					1·89	0·98	3·66	0·05
International guidelines								
No	1	–	–	–	–	–	–	–
Yes	1·65	0·95	2·85	0·07				
Pharmacists								
No	1	–	–	–	–	–	–	–
Yes	2·68	1·24	5·81	0·01*				
Health food stores								
No	–	–	–	–	1	–	–	–
Yes					3·71	1·08	12·64	0·03*
Attending campaigns on DS								
No	1	–	–	–	1	–	–	–
Yes	2·06	1·18	3·59	0·01*	1·309	0·69	2·48	0·41
Participating in research about DS								
No	–	–	–	–	1	–	–	–
Yes					2·107	1·02	4·34	0·04*
Knowledge score	–	–	–	–	0·971	0·93	1·00	0·08

DS, dietary supplement.

* A *P*-value less than 0 05 is considered statistically significant.

† Two separate regression models were used to identify variables associated with personal use of NS and HS, respectively.

### Practices regarding dietary supplements

The majority of the participants (81·8 %) stated that they discussed DS with their patients mostly tackling topics about their health benefits (80·5 %), side effects (69·0 %), usage (67·4 %) and toxicity (57·9 %). When asked if they sell DS, only few dietitians (5·3 %) reported that they did, and even fewer of them (2·2 %) reported formulating their own supplements.

Overall, 39·5 % of the dietitians reported recommending NS to their patients (Table 2) with vitamin D being the most recommended supplement (26·6 %) along with probiotics and prebiotics (24·8 %), Fe (24·1 %), Mg (21·9 %) and multivitamins (17·2 %) (see online Supplemental Table 3). On the other hand, dietitians were less inclined towards HS recommendation with only 16·6 % of the participants prescribing them in the intention of supplementing the diet. Among HS, the most recommended forms were ginger (10·3 %) and chamomile teas (8·8 %), along with green tea capsules (9·4 %) and ginger capsules (8·8 %) (see online Supplemental Table 4). Supplements were especially recommended for certain population groups including the elderly (43·3 %), pregnant and lactating women (41·7 %), adults (38·2 %), patients with chronic diseases (27·3 %) and children (11·6 %).

Dietitians mostly recommended DS to fill nutrient gaps (50·3 %), for skin/hair/nails health (41·5 %), gastrointestinal health (40·8 %), bone health (36·7 %) and overall health and wellness (36·1 %) and refrained from recommending them because of their concerns regarding supplements’ potential interactions with other treatments (19·8 %), their concerns regarding the negative effects (18·0 %) and regulation of DS (14·0 %).

Results of the multiple logistic regression for the factors associated with recommending DS to patients are presented in Table 5. Dietitians who personally used NS (OR = 3·38, *P* < 0·001), those who relied on pharmacists for information about DS (OR = 2·29, *P* = 0·01), and those who discussed DS with their patients (OR = 3·82, *P* = 0·01) were significantly more likely to recommend NS. Similarly, dietitians who personally used HS (OR = 12·23, *P* < 0·001), and those who discussed DS with patients (OR = 8·51, *P* = 0·01) were more likely to recommend them.

**Table 5:** Multiple logistic regression for the factors associated with recommending nutrient supplements (*n* 302) and herbal supplements (*n* 319) by dietitians to patients in Lebanon

	Nutrient supplements recommendation†				Herbal supplements recommendation			
	OR	Lower 95 % CI	Upper 95 % CI	*P*-value	OR	Lower 95 % CI	Upper 95 % CI	*P*-value
Age (years)	0·99	0·92	1·06	0·89	–	–	–	–
Years of practice	1·10	0·96	1·22	0·06	–	–	–	–
BMI (kg/m^2^)								
Overweight/obese (≥25)	1	–	–	–	–	–	–	–
Underweight (<18·5)	0·49	0·12	1·96	0·31				
Normal (18·5 to <25)	1·80	0·78	4·16	0·16				
Alcohol consumption								
Never	–	–	–	–	1	–	–	–
Occasionally					0·68	0·33	1·39	0·29
Using NS								
No	1	–	–	–	1	–	–	–
Yes	3·38	1·77	6·45	<0·01*	1·68	0·71	3·96	0·23
Using HS								
No	–	–	–	–	1	–	–	–
Yes					12·23	5·78	25·87	<0·01*
Sources of DS information								
Former trainings/workshops								
No	1	–	–	–	1	–	–	–
Yes	1·64	0·93	2·91	0·08	0·88	0·42	1·86	0·74
Pharmacists								
No	1	–		–	–	–	–	–
Yes	2·29	1·22	4·30	0·01*				
Health food stores								
No	–	–	–	–	1	–		–
Yes					1·00	0·22	4·57	0·99
Discussing DS with patients								
No	1	–	–	–	1	–	–	–
Yes	3·82	1·66	8·82	<0·01*	8·51	1·79	40·29	0·01*
Attending campaigns on DS								
No	1	–	–	–	–	–	–	–
Yes	1·27	0·73	2·22	0·39				

DS, dietary supplement; NS, nutrient supplement; HS, herbal supplement.

* A *P*-value less than 0·05 is considered statistically significant.

† Two separate regression models were used to identify variables associated with recommendation of NS and HS, respectively.

## Discussion

To our knowledge, this research was the first to explore the knowledge, personal use and practices of Lebanese dietitians regarding DS. Our results indicated that dietitians in Lebanon seemed to have good knowledge regarding DS. A higher knowledge score was associated with less years of experience, using scientific articles as source of DS information and participating in research on DS. Dietitians were predominantly users and prescribers of NS, with 70·8 % of them having personally used them and 39·5 % having recommended them to patients. While NS use was associated with being a non-smoker, prescribing NS was associated with NS use and DS discussion with patients. Only a minority of LD reported the use or prescription of HS, with HS use being associated with health food stores as a source of information and participating in research about DS. Whereas HS recommendation was associated with HS use and discussing DS with patients.

The mean knowledge score of Lebanese LD (60·5 %) was in line with the score of dietitians in South Africa (62·6 %) using the same survey instrument^(20)^, and comparable to that reported among dietitians in Iran (70 to 78 %)^(19)^ and the USA (75·1 %)^(33)^ despite the use of different assessment tools. This may be due to the fact that dietitians participating in these studies had similar fields of practice and were mostly consulting directly with patients. The similitude of dietetics curriculum in Lebanon and the USA may also reflect the similarity of knowledge levels between Lebanese and American dietitians. Contrary to the findings of other studies^(20,33)^, we found that LD with less years of experience were more knowledgeable about DS. We speculate that this might be attributable to the ability of younger and freshly graduated dietitians to recall more information concerning DS than older dietitians.

In terms of personal use, the prevalence of DS use among LD in Lebanon is similar to that of dietitians in the USA^(22,23,25)^, the Netherlands and Australia^(26)^ but is higher than that of dietitians in South Africa^(20)^ and Turkey^(34)^. As for HS, the prevalence of use was similar to dietitians in California^(23)^ but lower than dietitians in Oregon^(25)^, Massachusetts^(21)^ and Turkey^(34)^ which may be attributable to the lack of interest of Lebanese LD in HS as well as the lack of studies supporting the benefits of HS use, which may refrain dietitians from using them. Regarding the issue of cost, data shows that HS prices in Lebanon ranged from 20·000 to 600·000 Lebanese pounds per bottle at the time of the study, making these products expensive and even unaffordable by the average Lebanese. In addition, we postulate that dietitians may have underreported their use of HS as a result of their uncertainty regarding the classification of herbal products and whether they are considered as supplements or not, especially that DS classification in Lebanon is still unclear.

It is notable that LD in Lebanon mostly used DS to fill nutrient gaps which is consistent with the recommendations of the Academy of Nutrition and Dietetics^(35)^ but is not similar to results of previous studies conducted in the USA where dietitians primarily resorted to using DS to prevent or treat a health condition^(22,23,33)^. This may imply that the education of LD in Lebanon does not encourage dietitians to substitute physicians in treating health conditions but trains them to complement physicians’ activities in preventing, assessing and treating health problems.

In contrast with the finding of Cashman *et al.* among dietitians in Massachusetts, USA^(21)^, we did not find any association between knowledge score and the use of HS; however our results showed a positive association between HS use and participating in research about DS. As discussed, this may be due to the insufficient evidence supporting the efficacy of HS. Furthermore, our results pointed out that NS use was associated with attending campaigns and relying on pharmacists for DS information. This result was similar to that of a recent study^(36)^ highlighting the important role of Lebanese pharmacists in the education about CAM therapies where 88 % of CAM users stated that they can trust the information given by pharmacists. Moreover, Lebanese pharmacists’ positive beliefs regarding the efficacy of CAM products including DS^(37)^ may also have a huge influence on the promotion and encouragement of supplements’ use by dietitians.

It is also important to note that dietitians’ confidence about their DS education as well as their practices regarding DS may be affected by the absence of an official dietetic order in Lebanon which hampers the ability of dietitians to keep themselves up-to-date and receive refresher trainings and limits their scope of practice related to numerous topics including DS, in the absence of adequate regulations.

While the prevalence of DS recommendation in Lebanon is higher than that found in Australia^(26)^, it is significantly lower than that reported in South Africa^(20)^, USA^(22,23,25)^ and the Netherlands^(24)^. This might be possibly related to Lebanese dietitians’ concerns regarding potential interactions of DS with other treatments, their regulation in Lebanon and their negative effects that were all reported as main barriers to prescribing DS. Moreover, given the adherence to the Traditional Lebanese Mediterranean diet by the Lebanese population, dietitians in Lebanon may consider this dietary pattern as complete and nutritious, compared to a Western diet implicating lower quality^(38–40)^ and thus may not consider the prescription of DS to patients.

While previous research has shown that multivitamins, multi-minerals and Ca as a single mineral supplement were the most commonly recommended NS in the USA and South Africa^(20,23,33)^, our study found that the top three recommended NS by Lebanese LD were vitamin D, pre/probiotics and Fe. This result aligned with that of a study in the Netherlands where vitamin D was the most recommended NS^(24)^, and another in Iran where Fe-containing supplements were mostly recommended by dietitians^(19)^. It is well known that anaemia is prevalent among children (18 %)^(41)^, women of reproductive age (31·2 %) and pregnant women (35·2 %) in Lebanon^(42)^, and the incidence of vitamin D deficiency has been found to be high in the Middle Eastern countries, particularly in Lebanon^(43,44)^. Also, the prevalence of irritable bowel syndrome (IBS) has been well documented in Lebanese adults, being in the upper limit of worldwide prevalence (20 %)^(45,46)^. These results also justified why dietitians in this survey reported recommending DS to patients mainly for reasons related to filling nutrient gaps and improving gastrointestinal health.

Our results build on existing evidence^(23,24)^ that dietitians who indicated having used DS or discussed them with patients tended to recommend them more. Interestingly, the association of prescribing DS with years of practice was similar to that found by Lee *et al.* ^(25)^ and inconsistent with the other reported literature, where some studies showed that dietitians who had longer work experience were less likely to recommend any form of DS^(19,21,27)^. However, the mentioned studies did not account for potential confounding factors such as age, level of education, field of practice and personal use of DS. Furthermore, our study provided a new insight into the positive association between NS recommendation and having resorted to former workshops, having attended campaigns on DS and having relied on pharmacists for DS information.

### Strengths and limitations

Our research has many strengths, including the large number of participants selected from the MoPH list of all LD in Lebanon. Despite the large proportion of inaccessible dietitians (41·3 %), selection of participants using random sampling improved the representativeness of the sample. Another strength was the reminder message sent to the dietitians who accepted to participate and which played a role in the high participation rate (37·7 %), compared to other similar online surveys. However, the study was subject to some limitations including the questionnaire being self-reported, which may lead to response and social desirability bias, in addition to reporting and memory biases particularly in questions regarding the types of supplements used and prescribed over the last 12 months. Moreover, since it was an online survey, there was an inability to control the possible referral of dietitians to outside sources to answer knowledge questions. Future research could be conducted in different settings in aim to obtain a more reliable knowledge assessment. Finally, the questionnaire assessed micronutrients-related knowledge, and therefore it lacked questions aiming to determine dietitians’ HS knowledge which could be desirable for future research.

## Conclusion

From the findings of this study, we conclude that LD in Lebanon were generally more comfortable towards NS as opposed to HS, with the majority of them being knowledgeable about micronutrients. They were also more likely to use DS personally than to recommend them to their patients.

The growing use of DS among the general population and the reports of dietitians stating that they lack confidence and training in this area support to a great extent the need of further DS education, especially in regard to HS, by a greater incorporation of DS practices into dietetics curriculum and later into trainings and workshops. A proper education along with the elaboration of scientific guidelines regarding the use and recommendation practices of dietitians and the implementation of concise laws regarding regulations of DS, especially in Lebanon, would play a crucial role in supporting dietitians’ practices in regard to DS. Further research, specifically about HS, would be valuable to determine how education changes the way dietitians assess, monitor and evaluate their patients and improves the quality of patient care with respect to dietary supplementation.

## Supplementary material

For supplementary material accompanying this paper visit https://doi.org/10.1017/S136898002100495X. click here to view supplementary material
