# Supplementary material for: Knowledge, use and practices of licensed dietitians related to dietary supplements in Lebanon
Source: Public Health Nutr. 2021 Dec 22;25(7):1751–61. doi: 10.1017/S136898002100495X (PMC9991652; doi:10.1017/S136898002100495X)
Supplement: Supplementary file 1 [file S136898002100495Xsup001.docx]

**Supplementary Table 1. Types and frequencies of nutrient supplements used by dietitians in Lebanon.**

|  | Less than 1 time per week /  **Occasionally** | | **3-5 times per week/**  **Frequently** | | **Daily** | | **Total** | |
| --- | --- | --- | --- | --- | --- | --- | --- | --- |
|  | n | %† | **n** | **%**† | **n** | **%**† | **n** | **%*** |
| ***Multivitamins and minerals*** |  |  |  |  |  |  |  |  |
| Multivitamins + iron | 43 | 44.3 | 28 | 28.9 | 26 | 26.8 | 97 | 42.9 |
| B complex vitamins | 46 | 61.3 | 18 | 24.0 | 11 | 14.7 | 75 | 33.1 |
| Multivitamins minerals | 46 | 61.3 | 16 | 21.3 | 13 | 17.3 | 75 | 33.1 |
| Multivitamins alone | 47 | 71.2 | 12 | 18.2 | 7 | 10.6 | 66 | 29.2 |
| Multivitamins + calcium | 43 | 74.1 | 10 | 17.2 | 5 | 8.6 | 58 | 25.6 |
| ***Single vitamins*** | | | | | | | | |
| Vitamin D | 92 | 62.2 | 43 | 29.1 | 13 | 8.8 | 148 | 65.4 |
| Vitamin C | 53 | 51.5 | 37 | 35.9 | 13 | 12.6 | 103 | 45.5 |
| Vitamin B12 | 39 | 58.2 | 15 | 22.4 | 13 | 19.4 | 67 | 29.6 |
| Vitamin B6 | 42 | 63.6 | 14 | 21.2 | 10 | 15.2 | 66 | 29.2 |
| Vitamin B9 | 35 | 62.5 | 7 | 12.5 | 14 | 25 | 56 | 24.7 |
| Biotin | 40 | 78.4 | 5 | 9.8 | 6 | 11.8 | 51 | 22.5 |
| Vitamin E | 40 | 88.9 | 3 | 6.7 | 2 | 4.4 | 45 | 19.9 |
| Vitamin A | 37 | 84.1 | 5 | 11.4 | 2 | 4.5 | 44 | 19.4 |
| ***Single minerals*** |  | | | | | | | |
| Magnesium | 60 | 48.4 | 35 | 28.2 | 29 | 23.4 | 124 | 54.8 |
| Iron | 34 | 32.1 | 33 | 31.1 | 39 | 36.8 | 106 | 46.9 |
| Calcium | 42 | 66.7 | 11 | 17.5 | 10 | 15.9 | 63 | 27.8 |
| Zinc | 39 | 79.6 | 5 | 10.2 | 5 | 10.2 | 49 | 21.6 |
| Potassium | 38 | 95.0 | 0 | 0.0 | 2 | 5.0 | 40 | 17.6 |
| ***Other ingredients*** | | | | | | | | |
| Probiotics/Prebiotics | 61 | 65.6 | 19 | 20.4 | 13 | 14.0 | 93 | 41.1 |
| Omega 3/6 fatty acids | 58 | 68.2 | 18 | 21.2 | 9 | 10.6 | 85 | 37.6 |
| Nutritional yeast | 40 | 93.0 | 1 | 2.3 | 2 | 4.7 | 43 | 19.0 |
| Coenzyme Q10 | 40 | 97.6 | 1 | 2.4 | 0 | 0.0 | 41 | 18.1 |
| Glucosamine | 38 | 95.0 | 2 | 5.0 | 0 | 0.0 | 40 | 17.6 |
| Lycopene | 37 | 97.4 | 1 | 2.6 | 0 | 0.0 | 38 | 16.8 |
| Lutein | 37 | 97.4 | 1 | 2.6 | 0 | 0.0 | 38 | 16.8 |
| †Percentages were calculated based on the total number of participants who use each specific nutrient supplement.  *Percentage was calculated based on the total number of participants who use any nutrient supplement (n=226). | | | | | | | | |

|  | Less than 1 time per week/  **Occasionally** | | **3-5 times per week/**  **Frequently** | | **Daily** | | **Total** | |
| --- | --- | --- | --- | --- | --- | --- | --- | --- |
|  | n | %† | **n** | **%**† | **n** | **%**† | **n** | **%*** |
| ***Capsules*** |  |  |  |  |  |  |  |  |
| Green tea | 16 | 59.3 | 9 | 33.3 | 2 | 7.4 | 27 | 48.2 |
| Ginger | 14 | 56.0 | 10 | 40.0 | 1 | 4.0 | 25 | 44.6 |
| Curcumin/Turmeric | 9 | 64.3 | 5 | 35.7 | 0 | 0.0 | 14 | 25.0 |
| Ginseng | 11 | 84.6 | 2 | 15.4 | 0 | 0.0 | 13 | 23.2 |
| Flaxseed oil | 12 | 92.3 | 1 | 7.7 | 0 | 0.0 | 13 | 23.2 |
| Charcoal | 11 | 91.7 | 1 | 8.3 | 0 | 0.0 | 12 | 21.4 |
| Ginkgo | 10 | 90.9 | 1 | 9.1 | 0 | 0.0 | 11 | 19.6 |
| Bee pollen | 11 | 100 | 0 | 0.0 | 0 | 0.0 | 11 | 19.6 |
| Echinacea | 10 | 90.9 | 1 | 9.1 | 0 | 0.0 | 11 | 19.6 |
| Chicory | 9 | 100 | 0 | 0.0 | 0 | 0.0 | 9 | 16.0 |
| ***Teas*** | | | | | | | | |
| Green tea | 18 | 42.9 | 19 | 45.2 | 5 | 11.9 | 42 | 75.0 |
| Chamomile | 19 | 63.3 | 9 | 30.0 | 2 | 6.7 | 30 | 53.5 |
| Ginger | 17 | 56.7 | 12 | 40.0 | 1 | 30.3 | 30 | 53.5 |
| Peppermint | 11 | 45.8 | 11 | 45.8 | 2 | 8.3 | 24 | 42.8 |
| Moringa | 12 | 75.0 | 2 | 12.5 | 2 | 12.5 | 16 | 28.5 |
| Matcha | 13 | 92.9 | 1 | 7.1 | 0 | 0.0 | 14 | 25.0 |
| Hibiscus | 11 | 91.7 | 0 | 0.0 | 1 | 8.3 | 12 | 21.4 |
| †Percentages were calculated based on the total number of participants who use each specific herbal supplement.  *Percentage was calculated based on the total number of participants who use any herbal supplement (n=56). | | | | | | | | |

**Supplementary Table 2. Types and frequencies of herbal supplements used by dietitians in Lebanon.**

|  | Less than 1 time per week/  **Occasionally** | | **3-5times per week/**  **Frequently** | | **Daily** | | **Total** | |
| --- | --- | --- | --- | --- | --- | --- | --- | --- |
|  | n | %† | **n** | **%**† | **n** | **%**† | **n** | **%*** |
| ***Multivitamins and minerals*** |  |  |  |  |  |  |  |  |
| Multivitamins alone | 35 | 63.6 | 15 | 27.3 | 5 | 9.1 | 55 | 43.6 |
| Multivitamins + minerals | 29 | 56.9 | 14 | 27.5 | 8 | 15.7 | 51 | 40.4 |
| Multivitamins + iron | 29 | 58.0 | 15 | 30.0 | 6 | 12.0 | 50 | 39.6 |
| B complex vitamins | 27 | 58.7 | 14 | 30.4 | 5 | 10.9 | 46 | 36.5 |
| Multivitamins + calcium | 21 | 60.0 | 11 | 31.4 | 3 | 8.6 | 35 | 27.7 |
| ***Single vitamins*** |  | | | | | | | |
| Vitamin D | 46 | 54.1 | 28 | 32.9 | 11 | 12.9 | 85 | 67.4 |
| Vitamin C | 23 | 43.4 | 21 | 39.6 | 9 | 17.0 | 53 | 42.0 |
| Vitamin B12 | 29 | 58.0 | 12 | 24.0 | 9 | 18.0 | 50 | 39.6 |
| Vitamin B6 | 24 | 64.9 | 9 | 24.3 | 4 | 10.8 | 37 | 29.3 |
| Biotin | 22 | 64.7 | 7 | 20.6 | 5 | 14.7 | 34 | 26.9 |
| Vitamin B9 | 23 | 69.7 | 7 | 21.2 | 3 | 9.1 | 33 | 26.1 |
| Vitamin E | 23 | 79.3 | 5 | 17.2 | 1 | 3.4 | 29 | 23.0 |
| Vitamin A | 24 | 92.3 | 2 | 7.7 | 0 | 0.0 | 26 | 20.6 |
| ***Single minerals*** |  | | | | | | | |
| Iron | 33 | 42.9 | 20 | 26.0 | 24 | 31.2 | 77 | 61.1 |
| Magnesium | 38 | 54.3 | 18 | 25.7 | 14 | 20.0 | 70 | 55.5 |
| Zinc | 25 | 56.8 | 13 | 29.5 | 6 | 13.6 | 44 | 34.9 |
| Calcium | 24 | 60.0 | 7 | 17.5 | 9 | 22.5 | 40 | 31.7 |
| Potassium | 24 | 92.3 | 2 | 7.7 | 0 | 0.0 | 26 | 20.6 |
| ***Other ingredients*** |  | | | | | | | |
| Probiotics/Prebiotics | 31 | 39.2 | 34 | 43.0 | 14 | 17.7 | 79 | 62.6 |
| Omega 3/6 fatty acids | 34 | 50.7 | 23 | 34.3 | 10 | 14.9 | 67 | 53.1 |
| Glucosamine | 26 | 89.7 | 1 | 3.4 | 2 | 6.9 | 29 | 23.0 |
| Coenzyme Q10 | 24 | 85.7 | 2 | 7.1 | 2 | 7.1 | 28 | 22.2 |
| Nutritional yeast | 19 | 73.1 | 7 | 26.9 | 0 | 0.0 | 26 | 20.6 |
| Lycopene | 24 | 100 | 0 | 0.0 | 0 | 0.0 | 24 | 19.0 |
| Lutein | 22 | 100 | 0 | 0.0 | 0 | 0.0 | 22 | 17.4 |
| †Percentages were calculated based on the total number of participants who recommend each specific nutrient supplement.  *Percentage was calculated based on the total number of participants who recommend any nutrient supplement (n=126). | | | | | | | | |

**Supplementary Table 3. Types and frequencies of nutrient supplements recommended by dietitians to patients in Lebanon.**

|  | Less than 1 time per week/  **Occasionally** | | **3-5times per week/**  **Frequently** | | **Daily** | | **Total** | |
| --- | --- | --- | --- | --- | --- | --- | --- | --- |
|  | n | %† | **n** | **%**† | **n** | **%**† | **n** | **%*** |
| ***Capsules*** |  |  |  |  |  |  |  |  |
| Green tea | 8 | 26.7 | 17 | 56.7 | 5 | 16.7 | 30 | 56.6 |
| Ginger | 11 | 39.3 | 11 | 39.3 | 6 | 21.4 | 28 | 52.8 |
| Curcumin/Turmeric | 7 | 41.2 | 9 | 52.9 | 1 | 5.9 | 17 | 32.0 |
| Flaxseed oil | 10 | 71.4 | 4 | 28.6 | 0 | 0.0 | 14 | 26.4 |
| Ginseng | 10 | 83.3 | 1 | 8.3 | 1 | 8.3 | 12 | 22.6 |
| Echinacea | 5 | 55.6 | 1 | 11.1 | 3 | 33.3 | 9 | 16.9 |
| Charcoal | 6 | 85.7 | 0 | 0.0 | 1 | 14.3 | 7 | 13.2 |
| Ginkgo | 5 | 83.3 | 1 | 16.7 | 0 | 0.0 | 6 | 11.3 |
| Bee pollen | 4 | 66.7 | 2 | 33.3 | 0 | 0.0 | 6 | 11.3 |
| Chicory | 5 | 100 | 0 | 0.0 | 0 | 0.0 | 5 | 9.4 |
| ***Teas*** | | | | | | | | |
| Green tea | 8 | 19.0 | 22 | 52.4 | 12 | 28.6 | 42 | 79.2 |
| Ginger | 11 | 30.3 | 16 | 48.5 | 7 | 21.2 | 33 | 62.2 |
| Chamomile | 9 | 32.1 | 12 | 42.9 | 7 | 25.0 | 28 | 52.8 |
| Moringa | 7 | 35.0 | 10 | 50.0 | 3 | 15.0 | 20 | 37.7 |
| Peppermint | 8 | 44.4 | 7 | 38.9 | 3 | 16.7 | 18 | 33.9 |
| Matcha | 9 | 60.0 | 5 | 33.3 | 1 | 6.7 | 15 | 28.3 |
| Hibiscus | 4 | 66.7 | 1 | 16.7 | 1 | 16.7 | 6 | 11.3 |
| †Percentages were calculated based on the total number of participants who recommend each specific herbal supplement.  *Percentage was calculated based on the total number of participants who recommend any herbal supplement (n=53). | | | | | | | | |

**Supplementary Table 4. Types and frequencies of herbal supplements recommended by dietitians to patients in Lebanon.**
